# Circulating metabolites may illustrate relationship of alcohol consumption with cardiovascular disease

**DOI:** 10.1186/s12916-023-03149-2

**Published:** 2023-11-16

**Authors:** Yi Li, Mengyao Wang, Xue Liu, Jian Rong, Patricia Emogene Miller, Roby Joehanes, Tianxiao Huan, Xiuqing Guo, Jerome I. Rotter, Jennifer A. Smith, Bing Yu, Matthew Nayor, Daniel Levy, Chunyu Liu, Jiantao Ma

**Affiliations:** 1 Department of Biostatistics, School of Public Health, Boston University, Boston, MA USA; 2 Department of Neurology, School of Medicine, Boston University, Chobanian & Avedisian, Boston, MA USA; 3 Population Sciences Branch, National Heart, Lung, and Blood Institute, National Institutes of Health, Bethesda, MD USA; 4 Framingham Heart Study, Framingham, MA USA; 5 The Institute for Translational Genomics and Population Sciences, Department of Pediatrics, The Lundquist Institute for Biomedical Innovation at Harbor-UCLA Medical Center, Torrance, CA USA; 6 Department of Epidemiology, School of Public Health, University of Michigan, Ann Arbor, MI USA; 7 Department of Epidemiology, School of Public Health, Human Genetics, and Environmental Sciences, The University of Texas Health Science Center at Houston, Houston, TX USA; 8 Sections of Cardiovascular Medicine and Preventive Medicine and Epidemiology, Department of Medicine, Boston University School of Medicine, Boston, MA USA; 9 Nutrition Epidemiology and Data Science, Friedman School of Nutrition Science and Policy, Tufts University, Boston, MA USA

**Keywords:** Alcohol consumption, Metabolites, Cardiovascular disease

## Abstract

**Background:**

Metabolite signatures of long-term alcohol consumption are lacking. To better understand the molecular basis linking alcohol drinking and cardiovascular disease (CVD), we investigated circulating metabolites associated with long-term alcohol consumption and examined whether these metabolites were associated with incident CVD.

**Methods:**

Cumulative average alcohol consumption (g/day) was derived from the total consumption of beer, wine, and liquor on average of 19 years in 2428 Framingham Heart Study Offspring participants (mean age 56 years, 52% women). We used linear mixed models to investigate the associations of alcohol consumption with 211 log-transformed plasma metabolites, adjusting for age, sex, batch, smoking, diet, physical activity, BMI, and familial relationship. Cox models were used to test the association of alcohol-related metabolite scores with fatal and nonfatal incident CVD (myocardial infarction, coronary heart disease, stroke, and heart failure).

**Results:**

We identified 60 metabolites associated with cumulative average alcohol consumption (*p* < 0.05/211 ≈ 0.00024). For example, 1 g/day increase of alcohol consumption was associated with higher levels of cholesteryl esters (e.g., CE 16:1, beta = 0.023 ± 0.002, *p* = 6.3e − 45) and phosphatidylcholine (e.g., PC 32:1, beta = 0.021 ± 0.002, *p* = 3.1e − 38). Survival analysis identified that 10 alcohol-associated metabolites were also associated with a differential CVD risk after adjusting for age, sex, and batch. Further, we built two alcohol consumption weighted metabolite scores using these 10 metabolites and showed that, with adjustment age, sex, batch, and common CVD risk factors, the two scores had comparable but opposite associations with incident CVD, hazard ratio 1.11 (95% CI = [1.02, 1.21], *p* = 0.02) vs 0.88 (95% CI = [0.78, 0.98], *p* = 0.02).

**Conclusions:**

We identified 60 long-term alcohol consumption-associated metabolites. The association analysis with incident CVD suggests a complex metabolic basis between alcohol consumption and CVD.

**Supplementary Information:**

The online version contains supplementary material available at 10.1186/s12916-023-03149-2.

## Background

Alcohol drinking is a common lifestyle in many cultures and is a modifiable risk factor associated with over 200 health problems, including cardiovascular disease (CVD) [1], dementia [2], neuropsychiatric conditions [3], liver cirrhosis [4], diabetes [5], and several types of cancer, e.g., gastric cancer [6] and liver cancer [7]. For example, a systematic review and meta-analysis of 23 observational studies found that moderate alcohol consumption was related to a higher cardiovascular risk within 24 h after alcohol intake; however, after 24 h, moderate alcohol consumption seemed to have a protective effect on myocardial infraction [8]. In contrast, heavy alcohol intake had a continued risk for cardiovascular events [8].

Metabolites are small molecules that are intermediates or end-products of metabolism in many cellular processes [9, 10]. Metabolites can be quantified via high-throughput liquid chromatography with tandem mass spectrometry (LC/MS) methods [11]. Association analyses of alcohol consumption and metabolites may help us gain insights into the effect of alcohol consumption on disease pathways. Several European studies investigated associations of total alcohol intake with circulating concentrations of metabolites [12–14]. One study investigated the association of 123 metabolites with alcohol intake using a discovery (*n* = 1983) and replication (*n* = 991) study design in healthy participants. This study found that 72 metabolites were significantly associated with alcohol consumption in the discovery set, and 34 metabolites remained significance in the replication set [12]. A population-based study investigated 131 metabolites in 2090 individuals and revealed that 40 metabolites in men and 18 metabolites in women significantly differed in their concentrations between moderate-to-heavy and light alcohol drinking [14].

**Fig. 1:**
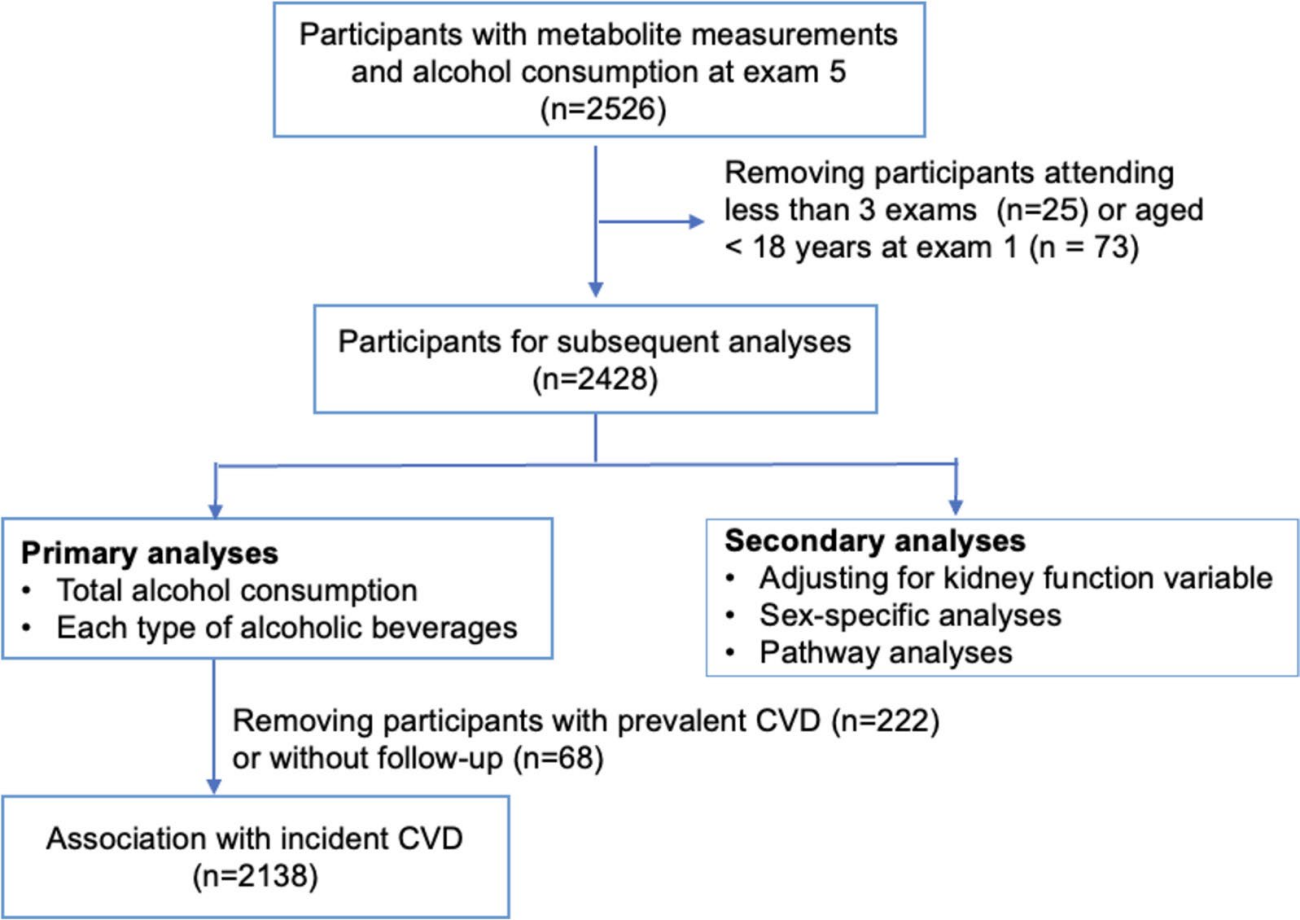
The study flowchart

While these metabolomic studies help us to understand the potential molecular basis of alcohol consumption, most studies analyzed the alcohol consumption measured at a single time point, which may not represent the habitual or long-term alcohol consumption. A better understanding of the relationships between long-term alcohol consumption and circulating metabolites, as well as the relationships of alcohol-associated metabolites with CVD risk, may help elucidate the complex relationship of alcohol consumption with etiology and progression of alcohol-associated diseases. To that end, our study used longitudinal data from the Framingham Heart Study (FHS) Offspring cohort to investigate the following three aims (Fig. 1). First, we conducted association analyses of cumulative average alcohol consumption over 20 years with circulating metabolites. Second, we analyzed the specific associations of metabolites with cumulative consumption of three types of alcoholic beverages: beer, wine, and liquor. Third, we conducted association analyses of alcohol-associated metabolites with incident CVD.

## Methods

### Population

The Offspring cohort (*n* = 5124) of the FHS was recruited in 1971, including the children of the Original cohort and spouses of these children [15]. The Offspring participants underwent in-person health examinations every 4 to 8 years to collect comprehensive demographic and clinical measures such as risk factors for cardiovascular and neurodegenerative diseases [16]. This study analyzed Offspring participants who had metabolite measurements at the fifth examination (*n* = 2526). We excluded participants who attended less than three examinations between examination 1 and examination 5 (*n* = 25) and those who were younger than 18 years old at examination 1 (*n* = 73). The final sample size was 2428 for statistical analyses (Fig. 1). All participants provided written informed consent. All FHS study protocols were approved by the Boston University Medical Center Institutional Review Board.

### Metabolite quantification

Targeted metabolite profiling was conducted by LC/MS platform and hydrophilic interaction chromatography method as previously described [17–19]. The targeted metabolite profiling includes negatively charged polar metabolites (i.e., organic acids, bile acids, and sugars), positively charged polar metabolites (i.e., amino acids, urea cycle intermediates, nucleotides), and lipid metabolite species (i.e., cholesterol esters (CE), diacylglycerols (DAGs), lysophosphatidylcholines (LPCs), lysophosphatidylethanolamines (LPEs), phosphatidylcholines (PCs), sphingomyelins (SMs), triacylglycerols (TAGs)) [17–20]. Two metabolites, TAG 54:5 and TAG 54:6, had identical values for all participants; we removed TAG 54:6 from the present analysis. Five metabolites were also excluded owing to large proportion of missingness in greater than 1000 participants, resulting in 211 metabolites for the subsequent analyses. Each metabolite measurement was natural log transformed and then standardized to have mean at zero and standard deviation (SD) at 1. Metabolite values beyond ± 4 SD away from the mean of that metabolite were set to be at ± 4 SD [19].

### Alcohol consumption

At each examination, alcohol consumption information was collected through the FHS technician-administered questionnaires [21]. The frequency of three types of alcoholic beverages (i.e., standard drinks of beer, wine, or liquor) in a typical week or month were collected. In this study, we used “grams of alcohol consumed per day”, which was derived from the summation of the total standard drinks of beer, wine (red or white), or liquor. One standard drink was defined as one 12 oz. beer, one 4 oz. wine, or one 1.5 oz. 80 proof alcohol spirit, and one drink contains approximately 14 g ethanol [21, 22]. We calculated the grams per day for all types of alcoholic beverages (i.e., total alcohol consumption), as well as for each type (i.e., grams per day of beer, wine, or liquor). To reflect long-term alcohol consumption, we calculated the cumulative average alcohol consumption, i.e., the mean over up to five examinations. We categorized participants into three groups according to their total average alcohol consumption: non-drinkers (0 g/day); moderate drinker (0.1–28 g/day in men and 0.1–14 g/day in women); and heavy drinkers (> 28 g/day in men and > 14 g/day in women).

### Disease traits

CVD events were identified through adjudication by a panel of three physicians [23]. The CVD event data were obtained from annual health history updates based on inpatient or outpatient medical records, physical examinations, and mortality registry. CVD was comprised of myocardial infarction (MI), coronary heart disease (CHD), stroke, heart failure (HF), and death to any cardiovascular conditions [24]. We analyzed incident CVD outcome after removal of the prevalent cases at the fifth examination.

### Covariates

Demographic information (sex and age), smoking status, dietary intake, time and intensity of physical activities, and medication use were obtained from standard questionnaire [25]. We calculated a physical activity index using the following formula: 1 × sleep hours/day + 1.1 × sedentary hours/day + 1.5 × slight activity hours/day + 2.4 × moderate activity hours/day + 5 × heavy activity hours/day. We used Dietary Approaches to Stop Hypertension (DASH) score to define diet quality. We calculated the diet quality score based on consumptions of eight dietary components: vegetables, fruits, nuts and legumes, whole grains, dairy, red and processed meat, sugar-sweetened beverages, and sodium [26]. Systolic blood pressure (SBP) was measured twice by physicians and the average blood pressure was used in the present analysis. Anthropometry was measured by technicians. Standard assays were used to determine levels of fasting glucose and plasma lipids. We defined diabetes if a participant had fasting glucose ≥ 126 mg/dL or using diabetes medications. All covariates were measured at the fifth examination.

### Statistical analysis

#### Association analysis of metabolites and alcohol consumption

In primary association analyses, we analyzed the relationships of metabolites (dependent variables) with cumulative average alcohol consumption of total alcohol, beer, wine, and liquor as independent variables (Fig. 1). We used linear mixed models to quantify these associations, adjusting for age, sex, (metabolite measurement) batch, smoking status, BMI, physical activity, and diet quality score as fixed effect covariates (all measured at the fifth examination), and familial relationship as a random effect covariate. We used Bonferroni correction (*p* < 0.05/211 $\approx$ 0.00024) to account for multiple hypothesis testing with 211 metabolites.

We conducted two secondary analyses as described below. The level of alcohol consumption is usually larger in men compared to women [27]; therefore, we tested sex–alcohol interaction by adding a production term of sex and alcohol consumption. We additionally adjusted for a serum creatinine-based estimated glomerular filtration rate (eGFR) to investigate if kidney function confounded the relationships between the cumulative average alcohol consumption and metabolites. (Fig. 1).

### Association analysis of alcohol-associated metabolites and CVD

Metabolites significantly associated with cumulative average alcohol consumption were used as independent variables in testing for associations with incident CVD. A Cox proportional hazards regression model was used, adjusting for sex, age, and batch in the base model. In the multivariable model, we additionally adjusted for BMI, SBP, hypertension treatment status, diabetes, smoking status, and total and high-density lipoprotein cholesterol levels. All covariates included were collected at the fifth examination.

To study an aggregate effect of metabolites with the development of CVD, we constructed weighted composite scores with CVD-associated metabolites that were identified in the base model. We used estimates from the association analyses between cumulative average alcohol consumption and the metabolites to weigh the concentrations of the corresponding metabolites. We then calculated metabolite scores as the linear combination of weighted metabolite concentrations: ${T}_{ij}={\sum }_{j}{W}_{j}{V}_{ij}$. Two metabolite scores were calculated based on the direction of the associations between alcohol consumption, metabolites, and incident CVD. The first score aggregated metabolites with consistent direction, i.e., those were either positively or negatively associated with both alcohol consumption and CVD. The second score aggregated metabolites with opposite direction, i.e., those were positively associated with alcohol consumption and negatively associated with CVD or those were negatively associated with alcohol consumption and positively associated with CVD. The composite scores were standardized with mean at 0 and standard deviation at 1. We performed association analysis of the continuous composite scores with the development of CVD, adjusting for age, sex, batch, BMI, SBP, hypertension treatment status, diabetes, smoking status, and total and high-density lipoprotein cholesterol levels. All association analyses were performed using R software (version 4.0.5).

### Pathway analysis for metabolites

Pathway analyses were performed to identify biological pathways related to the alcohol-associated metabolites using MetaboAnalyst 5.0 [28]. Two pathway libraries used in these analyses are Kyoto Encyclopaedia of Genes and Genomes (KEGG) and Small Molecule Pathway Database (SMPDB) for *Homo sapiens*. We conducted hypergeometric test with default parameters in MetaboAnalyst and reported significant pathways at false discovery rate (FDR) < 0.05.

## Results

### Participant characteristics

This study included mostly middle-aged to older community dwelling men and women (*n* = 2428, mean age = 55.9 $\pm$ 9.3 years, 52% women at fifth examination) (Table 1). The median of the cumulative average of total alcohol consumption was 7.7 g/day (interquartile range 16.8 g/day). Our participants were overweight (mean BMI 27.5 kg/m^2^) and 18.3% of them were current smokers at the fifth examination. Compared to women, men had greater alcohol consumption at all examinations: median consumption was 13.8 g/day in men versus 4.4 g/day in women at fifth examination. The pairwise Spearman correlation between total alcohol consumption at all five examinations is shown in Additional file 1: Fig. S1.

**Table 1:** Participant characteristics

Variable	Male (*n* = 1156)	Female (*n* = 1272)	Total (*n* = 2428)
Age, years	56.5 (9.2)	55.4 (9.4)	55.9 (9.3)
Body mass index, kg/m2	28.3 (4.1)	26.9 (5.5)	27.5 (4.9)
Physical activity index	35.8 (7.2)	33.3 (4.7)	34.5 (6.2)
Diet score	16.3 (4.3)	17.9 (3.9)	17.1 (4.1)
Estimated glomerular rate, ml/min/1.73 m2	73.8 (16.3)	67.5 (14.7)	70.5 (15.8)
Fasting blood glucose, mg/dL	105.3 (31.7)	97.9 (25.2)	101.4 (28.7)
Total cholesterol, mg/dL	203.0 (35.1)	209.7 (37.9)	206.5 (36.7)
High-density cholesterol, mg/dL	42.9 (11.1)	55.4 (15.4)	49.4 (14.9)
Systolic blood pressure, mmHg	129.7 (17.9)	124.2 (19.6)	126.9 (19.0)
Current smoker, *n* (%)	208 (18.0%)	236 (18.6%)	444 (18.3%)
Cumulative average alcohol consumption variable, g/day; median (Q1, Q3)			
Total alcohol consumption	13.8 (5.3, 27.5)	4.4 (1.7, 11.2)	7.7 (2.5, 19.3)
Beer consumption	4.4 (0.8, 13.4)	0 (0, 0.8)	0.8 (0, 5.2)
Wine consumption	1.3 (0.4, 4.0)	1.7 (0.4, 4.7)	1.3 (0.4, 4.3)
Liquor consumption	1.6 (0.4, 7.0)	1.0 (0.4, 3.2)	1.2 (0.4, 4.4)
Prevalent CVD	114 (12.5%)	78 (6.1%)	222 (9.1%)
Incident CVD	346 (29.9%)	290 (22.8%)	636 (26.2%)

The characteristics were measured at exam 5 (i.e., the baseline). Continuous variables are displayed as mean (standard deviation) or median (Q1–Q3). Binary variables are displayed as *n* (%)

### Associations of total alcohol consumption and metabolites

**Fig. 2:**
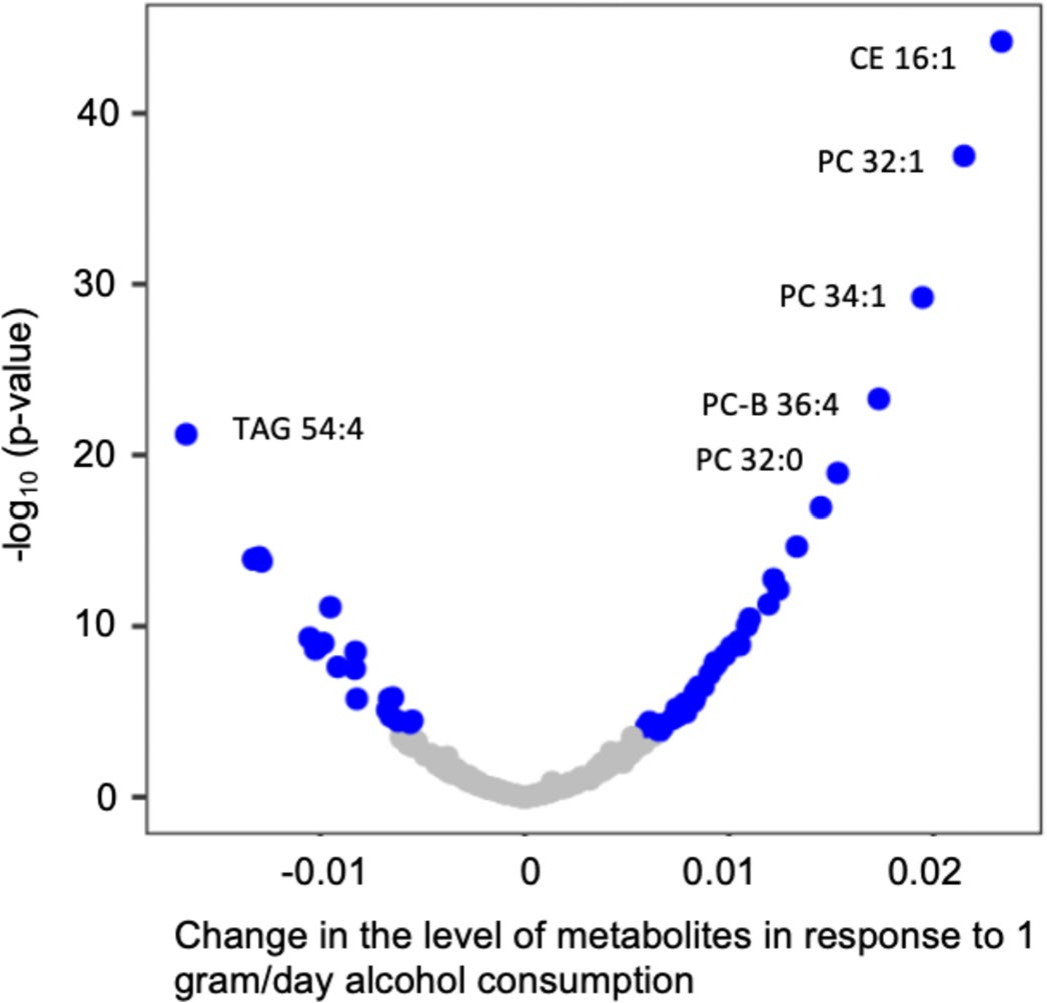
Volcano plot for association of cumulative average alcohol consumption and metabolites. Linear mixed model was performed adjusting for age, sex, batch, smoking status, BMI, physical activity index, and diet score as fixed effect, and family relationship as random effect. Blue dots represent significant relationship (*p* value < 0.00024), grey dots represent non-significant relationship. CE cholesteryl ester, PC phosphatidylcholine, TAG triacylglycerol

We found that 60 metabolites were significantly associated with the cumulative average total alcohol consumption (*p* < 0.05/211 $\approx$ 0.00024) adjusting for potential confounders (Table 2, Additional file 8: Table. S1). Of the 60 metabolites, 40 metabolites displayed positive associations with the cumulative average alcohol consumption (Fig. 2, Additional file 8: Table. S1). The most significant metabolite was cholesteryl palmitoleate (CE16:1), a plasma cholesteryl ester that is involved in cholesterol metabolism [29]. One gram per day higher alcohol consumption was associated with higher level of cholesteryl palmitoleate (beta = 0.023, *p* = 6.3e − 45) in the blood. Several phosphatidylcholine metabolites (e.g., PC 32:1 and PC 34:1) were positively associated with alcohol consumption, for example, 1 g/day higher of alcohol consumption was associated with higher level of PC 32:1 (beta = 0.021, *p* = 3.1e − 38) in blood. Among the 20 metabolites (Fig. 2) that were negatively associated with alcohol consumption, triacylglycerol 54:4 (TAG 54:4) displayed the most significant association (beta =  − 0.017, *p* = 6.17e − 22). With additional adjustment for eGFR, the association between alcohol consumption and metabolites remained largely the same (Pearson correlation coefficient = 0.99, Additional file 2: Fig. S2). Compared to the associations with the cumulative average total alcohol consumption, analysis using the current alcohol consumption at the fifth examination yielded similar alcohol–metabolite associations (Pearson *r* = 0.99; Additional file 3: Fig. S3).

**Table 2:** The top 20 significant metabolites associated with cumulative average alcohol consumption

Metabolites	Beta	SE	*P* value
CE 16:1	0.023	0.002	6.30E − 45
PC 32:1	0.021	0.002	3.10E − 38
PC 34:1	0.019	0.002	5.99E − 30
PC-B 36:4	0.017	0.002	5.18E − 24
TAG 54:4	− 0.017	0.002	6.17E − 22
PC 32:0	0.015	0.002	1.13E − 19
CE 20:5	0.014	0.002	1.14E − 17
LPC 20:5	0.013	0.002	2.17E − 15
TAG 52:4	− 0.013	0.002	9.05E − 15
TAG 54:5	− 0.013	0.002	1.18E − 14
TAG 52:3	− 0.013	0.002	1.58E − 14
TAG 48:0	0.012	0.002	1.74E − 13
Fumarate-malate	0.012	0.002	7.00E − 13
SM 24:1	0.012	0.002	5.17E − 12
Dimethylglycine	− 0.010	0.001	7.48E − 12
Xanthurenate	0.011	0.002	3.53E − 11
PC 34:4	0.011	0.002	9.52E − 11
TAG 52:5	− 0.011	0.002	4.81E − 10
PC 36:3	0.010	0.002	7.39E − 10

Top 20 out of 60 metabolites significantly associated with the cumulative average total alcohol consumption (*p* < 0.00024) adjusting for age, sex, batch, smoking status, BMI, physical activity index and diet score as fixed effect,  and family relationship as random effect

### Association of each type of alcoholic beverages and metabolites

**Fig. 3:**
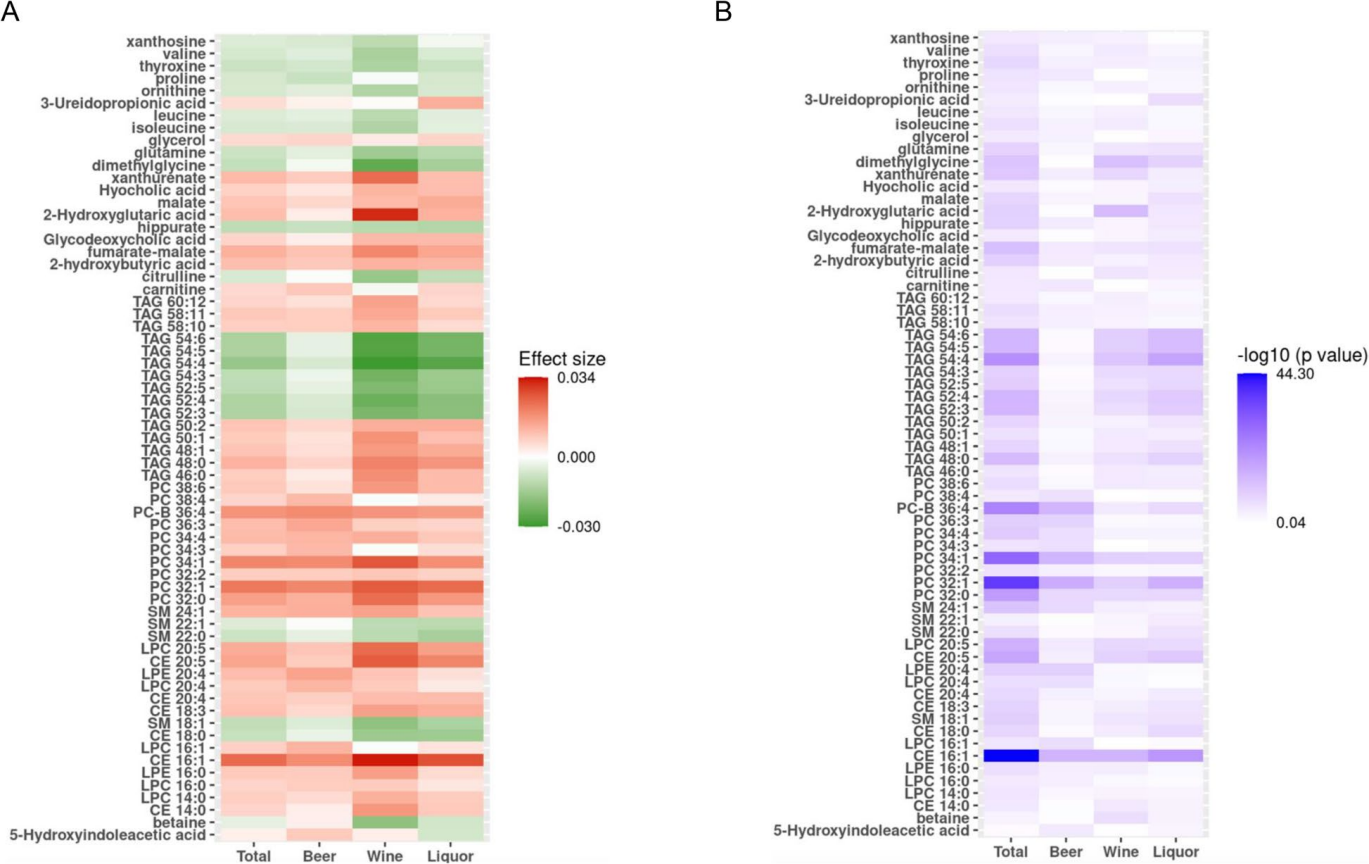
Comparison of association between different types of alcohol beverages. **A** Comparison of effect sizes between different types of alcohol beverages. **B** Comparison of -log 10 (*p* values) between different types of alcohol beverages. All linear mixed models adjusted for age, sex, batch, smoking status, BMI, physical activity index and diet score as fixed effect, and family relationship as random effect. CE cholesterol esters, DAG diacylglycerols, LPC lysophosphatidylcholines, LPE lysophosphatidylethanolamines, PC phosphatidylcholines, SM sphingomyelins, TAG triacylglycerols. Metabolites shown in panel had significant association with at least one type of alcohol (beer, wine, and liquor) or total alcohol consumption

We found that 19 metabolites were significantly associated with the cumulative average consumption of beer, 30 metabolites were significantly associated with the cumulative average consumption of wine, and 32 metabolites were significantly associated with the cumulative average consumption of liquor (Fig. 3, Additional file 8: Table. S1). Among the significant ones, seven metabolites (CE 16:1, LPC 20:5, PC 32:0, PC 32:1, PC 34:1, PC-B 36:4, and fumarate-malate) were significantly associated with the cumulative consumption of all three types of alcoholic beverages (Additional file 4: Fig. S4).

While the three types of alcoholic beverages displayed largely consistent associations with metabolites (Additional file 5: Fig. S5), beer consumption appeared to have slightly weaker associations compared to consumptions of wine and liquor (Additional file 5: Fig. S5). Among 50 metabolites associated with at least one type of alcohol consumption, we calculated the pair-wise ratio of regression coefficients for each metabolite. When we used an absolute ratio of two as cutoff, our analysis revealed that three metabolites (LPC 16:1, PC 34:3 and PC 38:4) displayed stronger positive association with the cumulative average consumption of beer; one metabolite (3-ureidopropionic acid) showed positive association with liquor consumption liquor; and three metabolites (betaine, 2-hydroxyglutaric acid, xanthurenate) were positively and two metabolites (isoleucine and valine) were negatively associated with wine consumption (Additional file 9: Table. S2).

### Sex-specific associations of metabolite with cumulative average total alcohol consumption

**Fig. 4:**
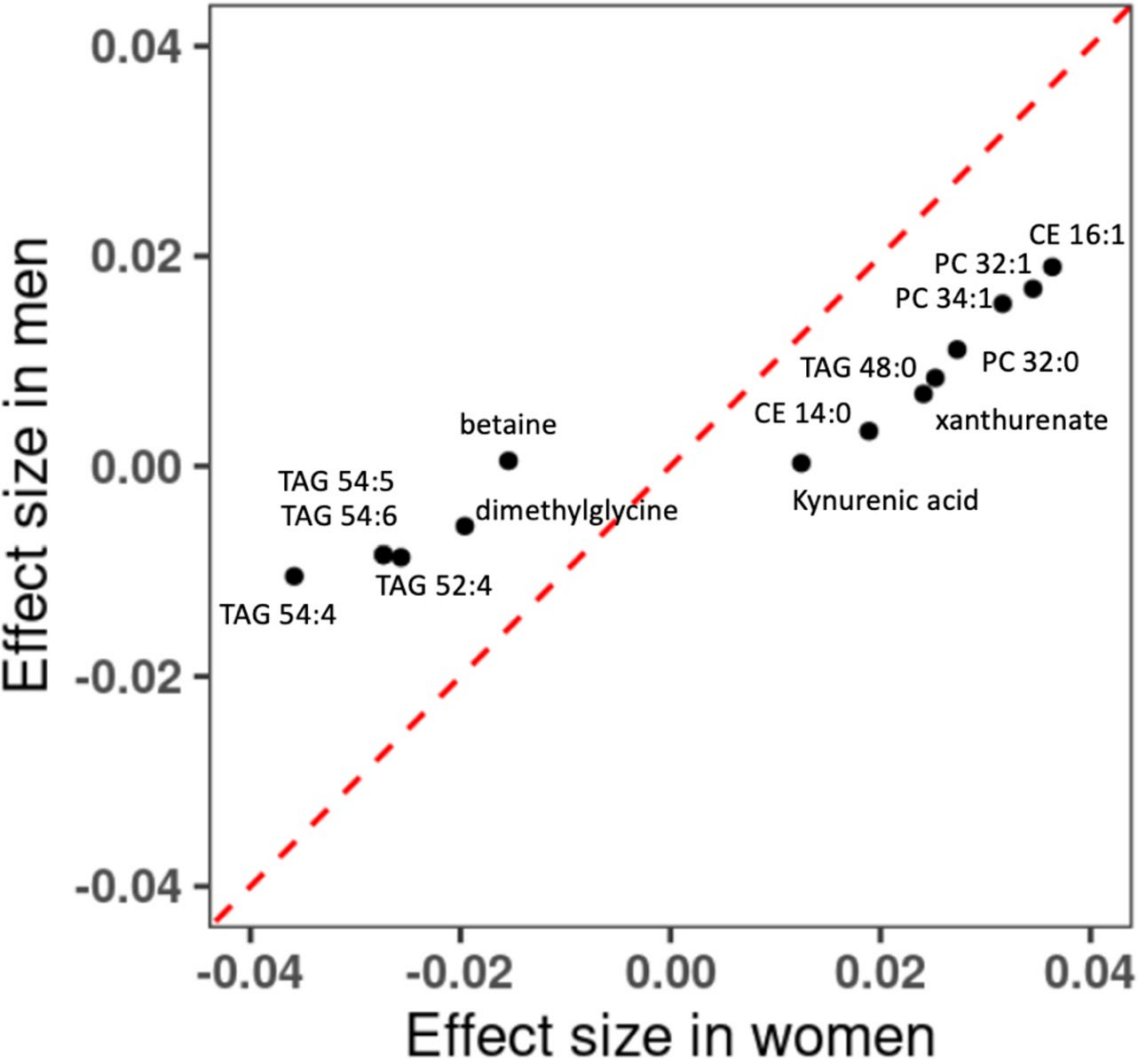
Comparison of associations of cumulative average alcohol consumption and metabolites between men and women. Linear mixed model was performed in women-only and men-only samples. Covariates included age, batch, smoking status, BMI, physical activity index and diet score as fixed effect, and family relationship as random effect. CE cholesteryl ester, PC phosphatidylcholine, TAG triacylglycerol

We conducted interaction test for sex and alcohol consumption and found that 13 metabolites (betaine, CE 14:0, CE 16:1, PC 32:0, PC 32:1, PC 34:1, TAG 48:0, TAG 52:4, TAG 54:4, TAG 54:5, xanthurenate, dimethylglycine, and kynurenic acid) had a significant interaction term (*p* < 0.05/211 = 0.00024). Eleven (CE 14:0, CE 16:1, PC 32:0, PC 32:1, PC 34:1, TAG 48:0, TAG 52:4, TAG 54:4, TAG 54:5, xanthurenate, and dimethylglycine) of these 13 metabolites were in 60 metabolites that were significantly associated with the cumulative average total alcohol consumption. We applied sex-specific analysis to examine the associations of cumulative average total alcohol consumption with these 13 metabolites. For women, the associations were significant for all these 13 metabolites (*p* < 0.05/13 = 0.0038), while for men, 10 metabolites (CE 16:1, PC 32:0, PC 32:1, PC 34:1, TAG 48:0, TAG 52:4, TAG 54:4, TAG 54:5, xanthurenate, and dimethylglycine) had significant associations (Additional file 10: Table. S3). The observed sex-alcohol interaction was due to the stronger associations in women than that in men (Fig. 4). For example, 1 g/day higher alcohol consumption was associated with 0.036 standard deviation increases in the level of CE 16:1 in women (*p* value = 7.26e − 25), while 0.019 in men (*p* value = 4.25e − 22).

### Pathway analysis for alcohol-associated metabolites

We conducted pathway analyses of the 60 metabolites that were significantly associated with the cumulative average total alcohol consumption. Using the KEGG library, we found three pathways to demonstrate evidence of enrichment among our significant metabolites including (1) arginine biosynthesis (FDR = 0.002); (2) valine, leucine, and isoleucine biosynthesis (FDR = 0.04); and (3) aminoacyl-tRNA biosynthesis (FDR = 0.007) (Additional file 11: Table. S4). Using the SMPDB library, we did not observe significant pathways at FDR 0.05 level (Additional file 12: Table. S5).

### Association with incident CVD

We included 2138 participants without prevalent CVD in association analyses of the 60 alcohol-associated metabolites with incident CVD. A total of 636 participants developed incident CVD with a median follow-up of 18.1 years. With adjustment for sex and age, alcohol consumption was not associated with incident CVD, hazard ratio (HR) = 1, *p* = 0.87. Additional adjustments for BMI, SBP, hypertension treatment status, diabetes, smoking status, and total and high-density lipoprotein cholesterol levels (i.e., multivariable model) did not substantially alter the association. In addition, we observed no significant pairwise comparisons in nondrinkers, moderate drinkers, and heavy drinkers. In the multivariable model, HR was 1.09 for nondrinkers vs. moderate drinkers (*p* = 0.62) and 1.15 for heavy drinkers vs. moderate drinkers (*p* = 0.16).

Ten metabolites (TAG 50:2, TAG 50:1, TAG 48:1, TAG 48:0, isoleucine, leucine, TAG 52:3, TAG 46:0, PC 32:1, and glutamine) were significantly associated with the development of CVD (*p* < 0.05/60 $\approx$ 0.0008) in the base model adjusting for age, sex, and batch (Additional file 13: Table. S6). Out of the ten metabolites, higher levels of nine metabolites (TAG 50:2, TAG 50:1, TAG 48:1, TAG 48:0, TAG 46:0, PC 32:1, TAG 52:3, isoleucine and leucine) and lower levels of one metabolite (glutamine) were associated with an increased risk of incident CVD (HR ranges from 1.16 to 1.33, *p* < 0.05/60≈0.0008). After additionally adjusting for BMI, SBP, hypertension treatment status, diabetes, smoking status, and total and high-density lipoprotein cholesterol level, the association with incident CVD remained nominally significant at *p* < 0.05 for four metabolites (TAG 50:2, TAG 50:1, TAG 48:1, and glutamine; Additional file 13: Table. S6). We further analyzed the associations of the 10 metabolites with the incidence of four CVD subtypes, including coronary heart disease (CHD), myocardial infarction (MI), heart failure (HF), and stroke. As shown in Additional file 14: Table. S7, with adjustment for age, sex, and batch, we observed that TAG 52:3 was associated with MI (HR = 1.36, *p* = 1.07e − 4), PC 32:1, TAG 48:0, TAG 50:1,TAG 50:2 and isoleucine were associated with HF (HR ranges from 1.27 to 1.40, *p* < 0.0008), TAG 48:1, TAG 50:1, TAG 50:2, TAG 52:3, isoleucine and leucine were associated with CHD (HR ranges from 1.23 to 1.32, *p* < 0.0008). However, for metabolites significantly related to CVD subtypes in the base model, additional adjustment for clinical risk factors attenuated the associations with CVD subtypes to nonsignificant (*p* > 0.05).

**Fig. 5:**
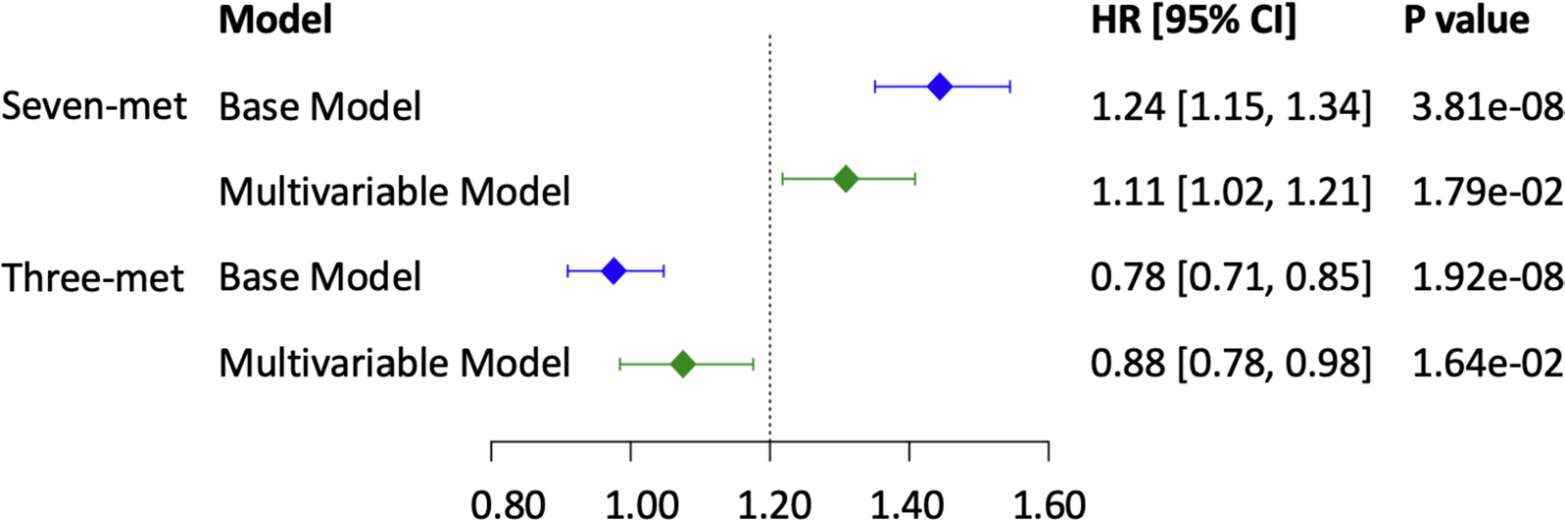
Forest plot for CVD with metabolite composite score. Seven-met, the weighted score obtained from seven metabolites, TAG 50:2, TAG 50:1, TAG 48:1, TAG 48:0, TAG 46:0, PC 32:1, and glutamine; three-met, the weighted score obtained from three metabolites, TAG 52:3, isoleucine, and leucine. Weights were obtained from the beta coefficients of association test between cumulative average alcohol consumption and metabolites in linear mixed models. In the Cox regression, base model included age, sex, and batch as covariates, and multivariable model additionally adjusted for BMI, SBP, hypertension treatment status, diabetes, smoking status, and total and high-density lipoprotein cholesterol level

We created two weighted metabolite scores; the first score included six metabolites (TAG 50:2, TAG 50:1, TAG 48:1, TAG 48:0, TAG 46:0 and PC 32:1) that had positive association with both the cumulative average total alcohol drinking and incident CVD in the base models and glutamine, which was negatively associated with both the cumulative average total alcohol drinking and incident CVD in the base model (Additional file 15: Table. S8). The second score included three metabolites (TAG 52:3, isoleucine and leucine), which had inverse association with the cumulative average total alcohol drinking and positive association with incident CVD in the base model (Additional file 15: Table. S8). In multivariable model analysis, per standard deviation increase of the first metabolite score was associated with 11% higher hazard of incident CVD (*p* = 0.02, 95%CI = [1.02, 1.21]; Fig. 5) and per standard deviation increase of the second metabolite score was associated with 12% lower hazard of incident CVD (*p* = 0.02, 95%CI = [0.78, 0.98]; Fig. 5).

### Nonlinear association of alcohol consumption with metabolites

We compared metabolite level among nondrinkers, moderate drinkers, heavy drinkers, adjusting for age, sex, batch, BMI, SBP, hypertension treatment status, diabetes, smoking status, and total and high-density lipoprotein cholesterol levels. At *p* < 0.05, we observed that for two metabolites (LPC 16:1 and histidine), their level in moderate drinkers was either lower or higher than both of that in nondrinkers and heavy drinkers (Additional file 6: Fig. S6). None of the two metabolites were associated with incident CVD.

## Discussion

In this present study, we investigated the associations between the cumulative average alcohol consumption and 211 circulating metabolites in the 2428 FHS participants. Of the 211 metabolites, the cumulative average alcohol consumption was associated with 60 metabolites. We found that nine metabolites were more strongly associated with a specific type of alcoholic beverage. We also found that the cumulative average total alcohol consumption displayed stronger associations with 13 metabolites in women than men. Furthermore, we created two alcohol consumption-associated metabolite scores and showed that they had comparable but opposite association with incident CVD. Taken together, with targeted metabolomic profiling, our study identified a series of alcohol consumption-associated circulating metabolites, and via these metabolites, alcohol consumption may have counteractive effects on CVD risk.

Our results showed that higher level of alcohol consumption was associated with higher plasma levels for about two-thirds of the 60 significant metabolites. Among the top positively associated metabolites were cholesteryl esters (e.g., CE16:1 and CE20:5), phosphatidylcholine (e.g., PC 32:1), and lysophosphatidylcholine (e.g., LPC 20:5). In addition, we observed that 14 plasma triacylglycerols (TAGs) were significantly associated with total alcohol consumption. Among alcohol-related TAGs, six (TAG 52:3, TAG 52:4, TAG 52:5, TAG 54:3, TAG 54:4, and TAG 54:5) displayed negative associations with alcohol consumption (i.e., lower alcohol consumption was associated with higher levels of TAGs) while eight (TAG 46:0, TAG 48:0, TAG 48:1, TAG 50:1, TAG 50:2, TAG 58:10, TAG 58:11, and TAG 60:12) displayed positive associations with alcohol consumption. TAGs emerge as biomarkers of a liver-to-β-cell axis that links hepatic β-oxidation to β-cell functional mass and insulin secretion in pancreas [30]. TAGs are broken into glycerol and free fatty acids in the process of lipolysis, and free fatty acids are either processed by beta-oxidation or converged to ketone [31]. In our association analyses between alcohol-associated metabolites and incident CVD, we showed that six TAGs (TAG 50:2, TAG 50:1, TAG 48:1, TAG 48:0, TAG 46:0, and TAG 52:3) were associated with incident CVD using the base models. Specifically, PC 32:1 and TAG 48:0 were positively associated with HF. In addition, TAG 52:3 was positively associated with both MI and CHD. Furthermore, TAG 50:1 and TAG 50:2 were positively associated with both HF and CHD. Among these six TAGs, the association remained significant (*p* < 0.05) with CVD for TAG 50:2, TAG 50:1, and TAG 48:1 after adjusting for common cardiometabolic CVD risk factors. However, likely due to the reduced number of cases for each CVD subtype (relative to the analysis using the composite incident CVD as the outcome variable), our multivariable analysis may lack sufficient statistical power. Therefore, we observed that additional adjustment for common cardiometabolic CVD risk factors attenuated associations with CVD subtypes (*p* > 0.05). Overall, these observations are in line with the well-known effects of alcohol intake on lipid metabolism [32].

Alcohol consumption was also associated with several types of circulating metabolites, other than TAGs. For example, we showed that alcohol consumption was associated with reduced levels of dimethylglycine. Dimethylglycine plays an important role in one-carbon metabolism as a methyl donor [33, 34]. This function may be related to our previous observations regarding the strong correlation of alcohol consumption with DNA methylation [22]. Nonetheless, the extent to which alcohol consumption affects the source of one-carbon metabolism and the subsequent impact on the risk of developing CVD warrant further investigation.

Among the metabolites that were negatively associated with alcohol consumption, valine, isoleucine, and leucine are branched-chain amino acids (BCAA). A recent review summarized the complex relationship between impaired BCAA homeostasis to CVD [35]. Several lines of evidence suggest that higher BCAA levels are associated with increased risk of obesity and diabetes, which is consistent with the present observations on the positive association of leucine and isoleucine with incident CVD. Nonetheless, future studies in larger sample size and diverse populations are needed to examine the relationships between alcohol consumption, BCAA, and CVD. Several lines of evidence suggest that higher BCAA levels are associated with increased risk of obesity and diabetes, e.g., Ho’s study demonstrated positive associations between BCAA levels and BMI, fasting glucose, Homeostatic Model Assessment for Insulin Resistance (HOMA-IR) in the same FHS participants [17]. These data are consistent with our previous findings on the inverse associations of alcohol consumption with obesity and type 2 diabetes [21], as well as the observations on the positive association of leucine and isoleucine with incident CVD in the present study. BCAAs are nitrogen donors for hepatic gluconeogenesis [36]. Our observations may support the notion that alcohol consumption, mainly moderate consumption, suppresses gluconeogenesis via lowering BCAA levels and subsequently controlling blood glucose to maintain normoglycemia. Nonetheless, future studies with larger sample sizes and diverse populations are needed to validate our observations, and experimental studies and clinical trials are needed to examine the relationships between alcohol consumption, BCAA, and CVD risk.

Our observations also highlight the complex relationship between alcohol consumption and circulating metabolites, which was demonstrated by the analysis using the two metabolite scores. Our observations suggest that, via circulating metabolites, alcohol drinking may have both positive and negative effects on CVD, and the two effects seemed to cancel each other out in our study samples. However, if certain factors disrupt the balance, it is possible that one effect may prevail over the other effect and leads to either increased or decreased risk of developing CVD. As such, future studies are warranted to understand what factors may modify the association of alcohol consumption and circulating metabolites, as well as their impact on the relationship of alcohol consumption with CVD development.

We observed that wine consumption and liquor had stronger associations with TAG, CE, and SM lipid metabolites, while beer had stronger associations with PC lipid metabolites. We also found that wine and liquor had different associations with amino acids, quinoline carboxylic acids, and hydroxy acids. Liquor consumption was significantly related to higher levels of 3-ureidopropionic acid, whereas wine consumption had stronger association with betaine, 2-hydroxyglutaric acid, xanthurenate, isoleucine, and valine compared to liquor. These observations suggest that consumption of different types of alcoholic beverages are associated with different metabolomic responses. However, the observed associations may also be driven by confounders such as unmeasured components in different alcoholic beverages or dietary and other environmental factors that were not adjusted for in the present analysis. Some of these metabolites such as betaine and isoleucine [35, 37] may play important roles in CVD development. Perhaps due to the short list of metabolites examined, the present study did not support the notion that a certain type of alcohol may bring specific benefits to reduce CVD risk. Future studies including a comprehensive list of metabolites are warranted to investigate this issue.

It was found by van Roekel et al. that total alcohol intake was associated with 34 circulating metabolites, including 3 acylcarnitines, the amino acid citrulline, 4 lysophosphatidylcholines, 13 diacylphosphatidylcholines, 7 acyl-alkylphosphatidylcholines, and 6 sphingomyelins [12] among middle-aged (mean age = 58.3) participants in European Prospective Investigation into Cancer and Nutrition Cohort. Among the 34 metabolites significant in the van Roekel’s study, 13 metabolites were also measured by our metabolite platforms, including LPC 16:0, LPC 16:1, LPC 20:4, PC 32:0, PC 32:1, PC 32:2, PC 34:1, PC 34:3, PC 34:4, PC-B 36:4, PC 38:6, PC 32:1, and SM 24:1. All of the 13 metabolites were significant in our analysis for total alcohol consumption (Additional file 7: Fig. S7, Additional file 16: Table. S9).

In a cross-sectional analysis, Würtz et al. examined the association of alcohol intake with 76 metabolites among young adults (aged 25–45) [38]. In that study, 36 metabolites were considered significantly associated with alcohol consumption. Of these 36 metabolites, 11 metabolites (glutamine, glycine, alanine, isoleucine, leucine, valine, lactate, pyruvate, glycerol, citrate, and creatinine) were also included in the present study, and 3 of 11 metabolites (glutamine, glycerol and leucine) reached significance using our data (Additional file 17: Table. S10). Glutamine and glycerol displayed similar association across the two cohorts. Interestingly, compared to our findings, the Würtz et al. study showed an opposite association between total alcohol consumption and leucine. Leucine is one member of the second metabolite score in the present study, which was inversely associated with incident CVD. As such, this observation may echo our abovementioned hypothesis that certain unknown factor(s) may modify the alcohol–metabolite association in different study samples. In addition, six metabolites (glutamine, glycine, alanine, isoleucine, leucine, valine) were included in all three studies (the Würtz et al. study, the van Roekel’s study, and the present study). Four of the six common metabolites were significant in our study and three of them were significant in Würtz et al.’s study, but none of them were significant in van Roekel’s study (Additional file 17: Table. S10). Together, these observations highlight the need for future studies to comprehensively investigate the heterogeneity with respect to alcohol–metabolite associations in different populations using harmonized metabolite panels.

We observed that, for 13 metabolites, their association strength with alcohol consumption was stronger in women compared to that in men. Women generally have smaller body sizes; consuming the same amount of alcohol would end up with a higher blood alcohol concentration for an average woman compared to an average man. In addition, women may have greater ethanol clearance than men, given the same lean body mass [39]. This may, at least partly, explain our observation with respect to the stronger metabolite response in women. Whether the observed sex–alcohol interaction can be affected by other factors, as well as its impact on CVD and clinical outcomes, may need future investigations.

### Strengths and limitations of the study

Our study had several strengths. The most important advantage was that alcohol drinking data from five exams across around 20 years were utilized in the current study. Our study had several limitations. Because of the observational nature of our findings and no experimental validation, causality cannot be inferred. Despite that the sample size in our study was large, the population was primarily white, middle-aged participants. Therefore, the findings from our study may not be generalizable to populations of different races and age groups. As shown in Additional file 1: Fig. S1, cumulative average consumption is a good proxy for long-term alcohol consumption. However, the analysis using total alcohol consumption at exam 5 yielded a similar alcohol–metabolite association. This observation may be driven by the possibility that many of our study participants maintained a low-to-moderate alcohol drinking habit. Future studies are needed to validate our findings in other populations with longitudinal alcohol measurements. Alcohol consumption was measured by questionnaires and calculated based on standard serving size. This approach is cost-effective; however, measurement errors may bias the observed association between alcohol intake and metabolite levels. We acknowledge that the metabolites data analyzed in our study are comprised of a targeted panel. Association between alcohol drinking and untargeted metabolites remained to be studied. Research utilizing metabolite platforms including those from exogenous sources, e.g., ethyl glucuronide and derivates of resveratrol, are needed to better understand the alcohol–metabolite relationship. We observed that sex may modify the association between total alcohol consumption and metabolite levels. Several other factors may also modify the observed associations. For example, the presence or absence of food in the stomach can change the rate of alcohol absorption and metabolism [40, 41] and subsequently affect the association of alcohol consumption with circulating metabolite profiles. In addition, the use of average alcohol consumption in the present analysis may not reflect participants’ diverse drinking patterns. For example, the information on alcohol consumption patterns such as drinking alcohol with or without meals or weekend binge drinking was not collected. Participants’ genetic background is a key factor that may modify alcohol metabolism [42], which may need further studies with larger sample size.

## Conclusions

The present study identified a series of alcohol-associated circulating metabolites. Our observations suggest that, via some metabolites, alcohol consumption may have counteractive effects on CVD. Future studies are required to validate our findings and to investigate factors that may modify the associations between alcohol consumption and metabolite levels, particularly for metabolites that potentially contribute to CVD risk, in larger and diverse study samples.

### Supplementary Information

**Additional file 1: Fig.S1. **The Pairwise Spearman Correlation Coefficient between Total Alcohol Consumption at All Five FHS Exams.

**Additional file 2: Fig. S2. **Comparison of Association of Alcohol Consumption and Metabolites with or without Adjusting for eGFR. Panel A: comparison of beta; Panel B: comparison of -log10 (p value). Two models both were performed adjusting for age, sex, batch, smoking status, BMI, physical activity index and diet score as fixed effect, and family relationship as random effect. eGFR, estimated glomerular filtration rate.

**Additional file 3: Fig. S3. **Comparison with Association of Alcohol Consumption at Exam5 and Metabolites. Panel A: comparison of beta; Panel B: comparison of -log10 (p value). Two models both were performed adjusting for age, sex, batch, smoking status, BMI, physical activity index and diet score as fixed effect, and family relationship as random effect.

**Additional file 4: Fig. S4. **Number of Metabolites Significantly Associated with Each Type of Alcohol Consumption. Panel A: three type of alcohol consumption; Panel B: three type of alcohol consumption and total alcohol consumption. All models were performed adjusting for age, sex, batch, smoking status, BMI, physical activity index and diet score as fixed effect, and family relationship as random effect.

**Additional file 5: Fig. S5. **Comparison of Association Analyses of Alcohol Consumption with Metabolites. Panel A-C, comparison of effect size from association analyses of cumulative average total alcohol consumption and each type of alcohol consumption with metabolites. Panel D-F, comparison of effect size from association analyses each type of alcohol consumption with metabolites. All models were performed adjusting for age, sex, batch, smoking status, BMI, physical activity index and diet score as fixed effect, and family relationship as random effect. The pairwise Pearson correlation coefficients of regression coefficients was 0.61, 0.64, and 0.88 for beer vs. wine, beer vs. liquor, and wine vs. liquor.

**Additional file 6: Fig. S6. **Comparisons between nondrinkers, Moderate drinkers, and heavy drinkers. Values are regression coefficients and 95% confidence interval calculated using moderate drinkers as reference. Models were adjusted for age, sex, batch, smoking status, BMI, physical activity index and diet score as fixed effect, and family relationship as random effect.

**Additional file 7: Fig. S7. **Comparison to Roekel’s Study. Panel A: comparison of beta; Panel B: comparison of t value. Only in this analysis, alcohol consumption (g/day) in this study was plus 1 and natural log transformed. The sex and batch-adjusted residual of metabolites from linear mixed model were used as outcome. Then we applied linear mixed model for alcohol drinking and residual of metabolites in linear mixed model, adjusting for age, sex, smoking status, BMI, physical activity, diet score as fixed effect, and familial relationship as random effect. But Roekel’s study used linear model and covariates included age at blood collection, sex, country, fasting status at blood collection, smoking status, BMI, Cambridge physical activity index, and daily intake of energy, meat and meat products, fish, and shellfish.

**Additional file 8: Table. S1. **Association between Cumulative Average Alcohol Consumption and Metabolites. Total, total amount of alcohol consumption; beer, beer consumption; wine, wine consumption; liquor, liquor consumption. These alcohol consumptions were average consumption across exams 1 to 5. Linear mixed models were performed adjusting for age, sex, batch, smoking status, BMI, physical activity index and diet score as fixed effect and family relationship as random effect.

**Additional file 9: Table. S2. **Comparison between Each Type of Cumulative Average Alcohol Consumption and Metabolites. Total, total amount of alcohol consumption; beer, beer consumption; wine, wine consumption; liquor, liquor consumption. These alcohol consumptions were average consumption across exams 1 to 5. Linear mixed models were performed adjusting for age, sex, batch, smoking status, BMI, physical activity index and diet score as fixed effect and family relationship as random effect.

**Additional file 10: Table. S3. **Association between Cumulative Average Alcohol Consumption and Metabolites by Sex. Linear mixed models were performed adjusting for age, batch, smoking status, BMI, physical activity index and diet score as fixed effect and family relationship as random effect.

**Additional file 11: Table. S4. **Pathway Analysis Results for 60 Metabolites Significantly Associated with Total Alcohol Consumption under KEGG Library.

**Additional file 12: Table. S5. **Pathway Analysis Results for 60 Metabolites Significantly Associated with Total Alcohol Consumption under SMPDB Library.

**Additional file 13: Table. S6. **Association between Incident CVD and alcohol-related Metabolites. Base models were performed adjusting for age, sex and batch. Multivariable models were performed additionally adjusting for BMI, SBP, hypertension treatment status, diabetes, smoking status, total and high-density lipoprotein cholesterol level.

**Additional file 14: Table. S7. **Association between Incident CVD subtypes and alcohol-related Metabolites. Base models were performed adjusting for age, sex and batch. Multivariable models were performed additionally adjusting for BMI, SBP, hypertension treatment status, diabetes, smoking status, total and high-density lipoprotein cholesterol level.

**Additional file 15: Table. S8. **Beta Direction from Association between Cumulative Average Alcohol Consumption and Metabolites, Association between Incident CVD and alcohol-related Metabolites.

**Additional file 16: Table. S9. **Comparison to Van Roekel's Study. Only in this analysis, our alcohol consumption (g/day) was plus 1 and natural log-transformed. The sex and batch-adjusted residual of metabolites from linear mixed model were used as outcome. Linear mixed models were performed for alcohol drinking and residual of metabolites in linear mixed model, adjusting for age, sex, smoking status, BMI, physical activity, diet score as fixed effect, and familial relationship as random effect.  Refer to reference 12 for details in Van Roekel's study.

**Additional file 17: Table. S10. **Comparison to Würtz’s study. Refer to reference 38 for detail statistical analysis in the Wurtz’s study, and significant threshold in their study is 0.0016. And reference 12 for details in Van Roekel's study, and FDR *p* value<0.05 was significance level.

## Data Availability

The Framingham Heart Study datasets analyzed in the present study are available at the dbGAP Study Accession: phs000007.v33.p14 (https://www.ncbi.nlm.nih.gov/projects/gap/cgi-bin/study.cgi?study_id=phs000007.v33.p14) via the Controlled Access Data.

## Abbreviations

CE: Cholesterol ester
CHD: Coronary heart disease
CVD: Cardiovascular disease
DAG: Diacylglycerol
DASH: Dietary Approaches to Stop Hypertension
eGFR: Estimated glomerular filtration rate
FDR: False discovery rate
FHS: Framingham Heart Study
HF: Heart failure
HR: Hazard ratio
KEGG: Kyoto Encyclopaedia of Genes and Genomes
LC/MS: Liquid chromatography with tandem mass spectrometry
LPC: Lysophosphatidylcholine
LPE: Lysophosphatidylethanolamine
MI: Myocardial infarction
PC: Phosphatidylcholine
SBP: Systolic blood pressure
SD: Standard deviation
SE: Standard error
SM: Syphingomyelin
SMPDB: Small Molecule Pathway Database
TAG: Triacylglycerol
