# Supplementary figures and images for: Circulating metabolites may illustrate relationship of alcohol consumption with cardiovascular disease
Source: BMC Med. 2023 Nov 16;21:443. doi: 10.1186/s12916-023-03149-2 (PMC10652547; doi:10.1186/s12916-023-03149-2)

**Additional file 1: Fig.S1**


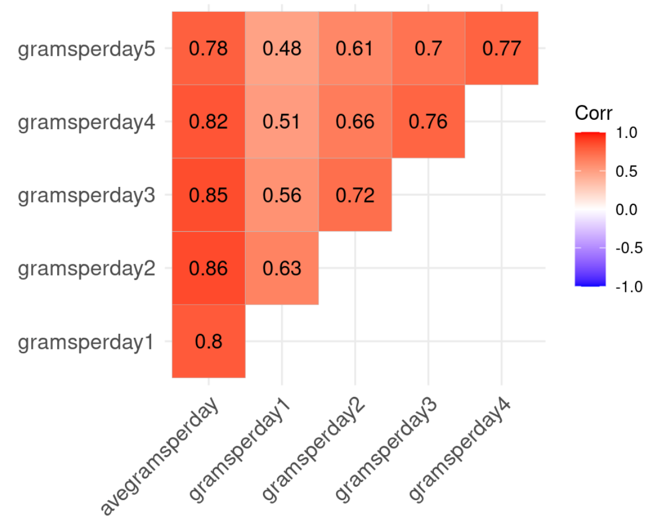

Supplement: Supplementary file 1 — Additional file 1: Fig.S1. The Pairwise Spearman Correlation Coefficient between Total Alcohol Consumption at All Five FHS Exams. [file 12916_2023_3149_MOESM1_ESM.docx]

**Additional file 2: Fig.S2**

**A B**


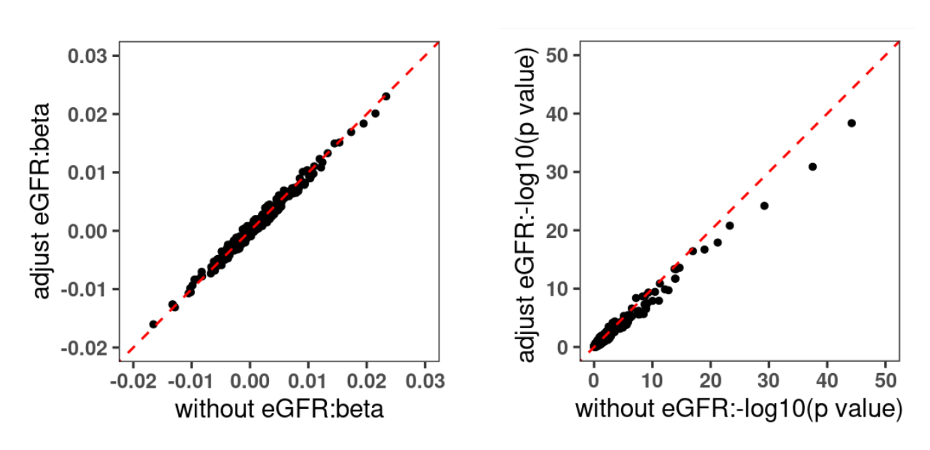

Supplement: Supplementary file 2 — Additional file 2: Fig. S2. Comparison of Association of Alcohol Consumption and Metabolites with or without Adjusting for eGFR. Panel A: comparison of beta; Panel B: comparison of -log10 (p value). Two models both were performed adjusting for age, sex, batch, smoking status, BMI, physical activity index and diet score as fixed effect, and family relationship as random effect. eGFR, estimated glomerular filtration rate. [file 12916_2023_3149_MOESM2_ESM.docx]

**Additional file 3: Fig.S3**

**A B**


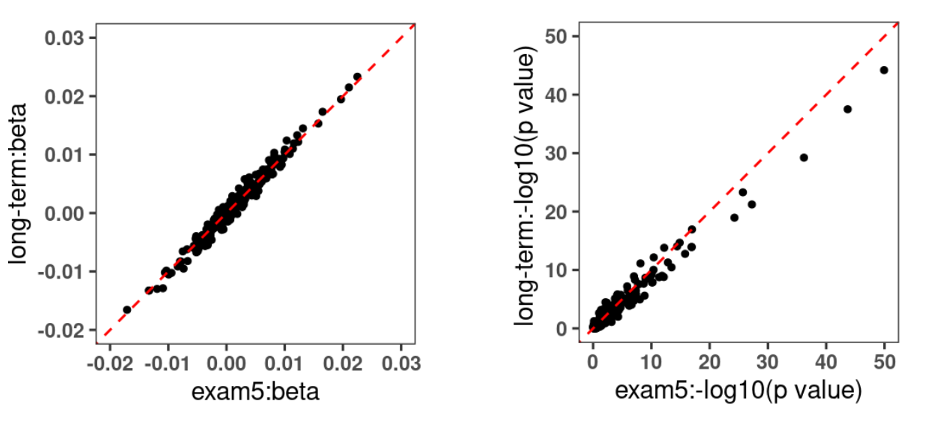

Supplement: Supplementary file 3 — Additional file 3: Fig. S3. Comparison with Association of Alcohol Consumption at Exam5 and Metabolites. Panel A: comparison of beta; Panel B: comparison of -log10 (p value). Two models both were performed adjusting for age, sex, batch, smoking status, BMI, physical activity index and diet score as fixed effect, and family relationship as random effect. [file 12916_2023_3149_MOESM3_ESM.docx]

**Additional file 4: Fig.S4**


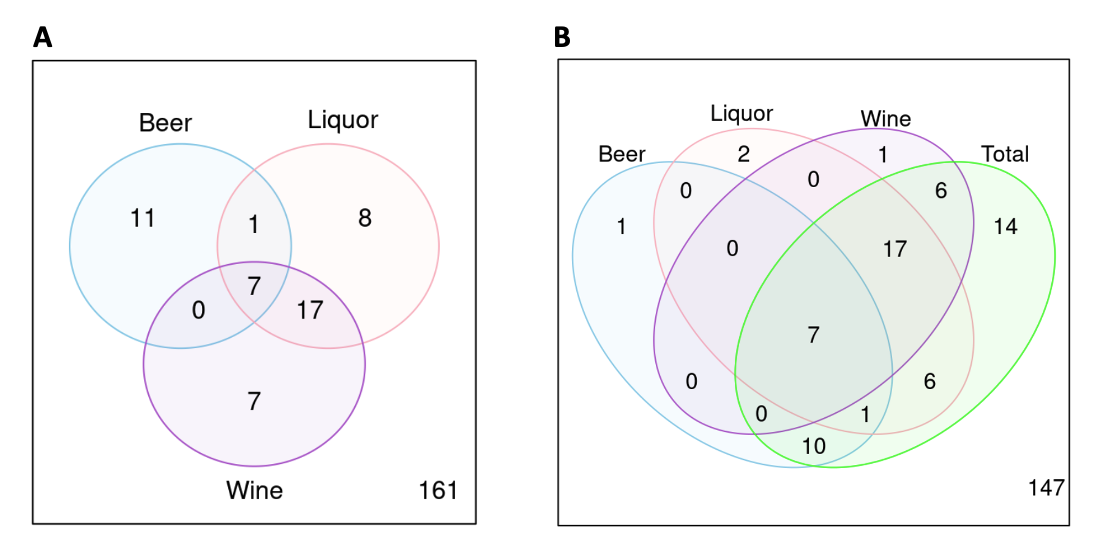

Supplement: Supplementary file 4 — Additional file 4: Fig. S4. Number of Metabolites Significantly Associated with Each Type of Alcohol Consumption. Panel A: three type of alcohol consumption; Panel B: three type of alcohol consumption and total alcohol consumption. All models were performed adjusting for age, sex, batch, smoking status, BMI, physical activity index and diet score as fixed effect, and family relationship as random effect. [file 12916_2023_3149_MOESM4_ESM.docx]

**Additional file 5: Fig.S5**


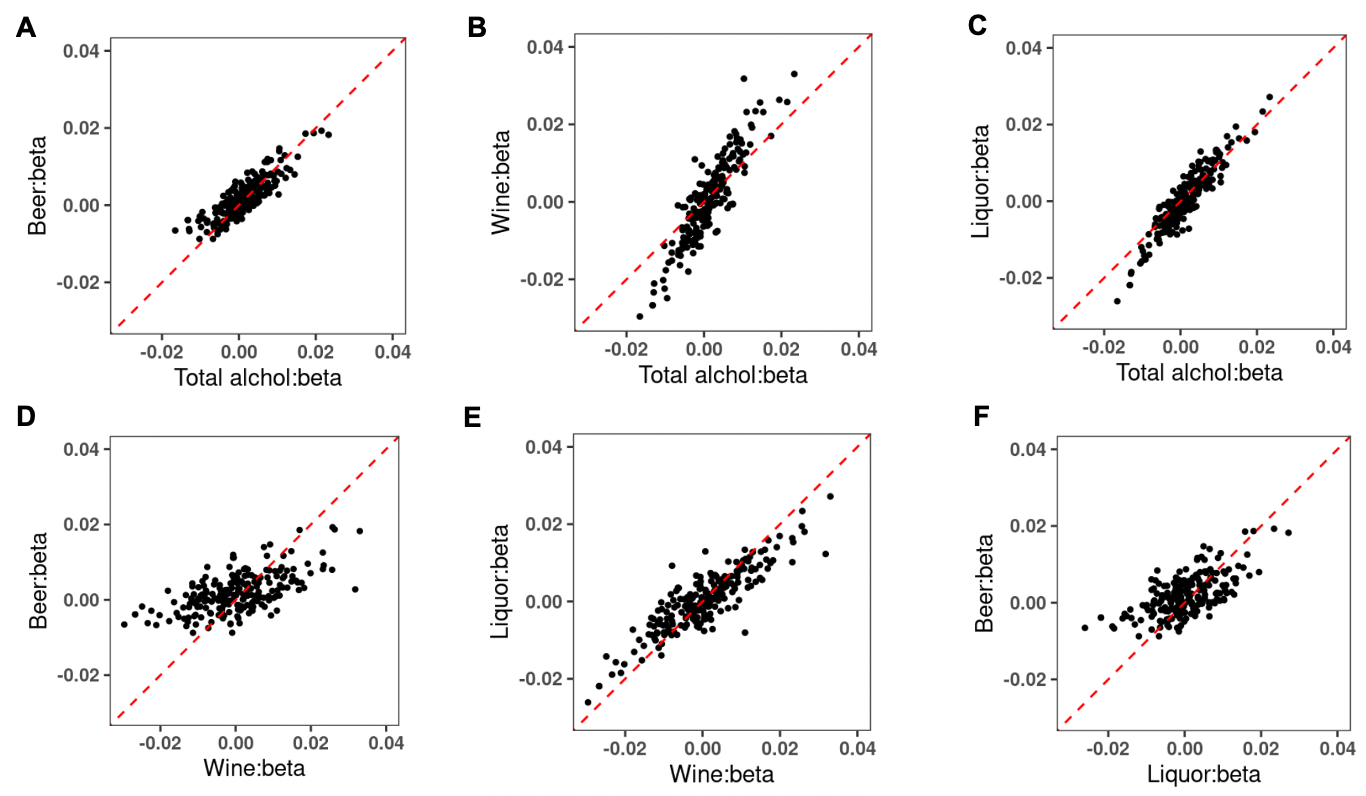

Supplement: Supplementary file 5 — Additional file 5: Fig. S5. Comparison of Association Analyses of Alcohol Consumption with Metabolites. Panel A-C, comparison of effect size from association analyses of cumulative average total alcohol consumption and each type of alcohol consumption with metabolites. Panel D-F, comparison of effect size from association analyses each type of alcohol consumption with metabolites. All models were performed adjusting for age, sex, batch, smoking status, BMI, physical activity index and diet score as fixed effect, and family relationship as random effect. The pairwise Pearson correlation coefficients of regression coefficients was 0.61, 0.64, and 0.88 for beer vs. wine, beer vs. liquor, and wine vs. liquor. [file 12916_2023_3149_MOESM5_ESM.docx]

**Additional file 6: Fig.S6**

**
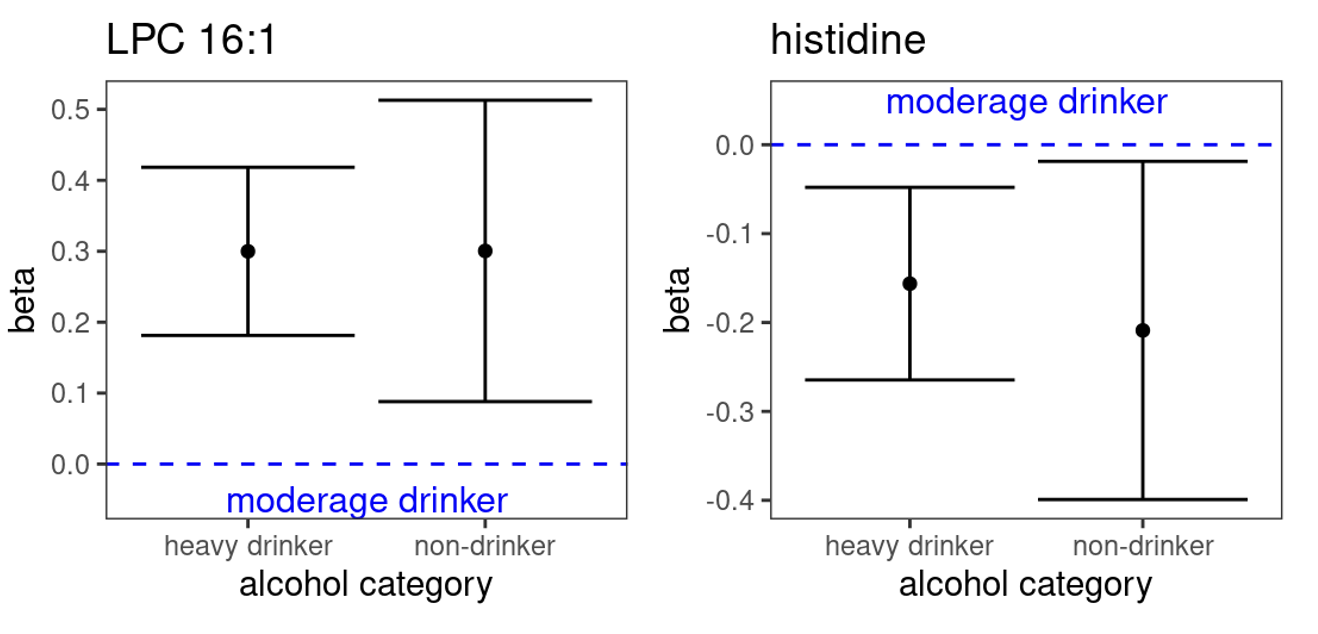
**

Supplement: Supplementary file 6 — Additional file 6: Fig. S6. Comparisons between nondrinkers, Moderate drinkers, and heavy drinkers. Values are regression coefficients and 95% confidence interval calculated using moderate drinkers as reference. Models were adjusted for age, sex, batch, smoking status, BMI, physical activity index and diet score as fixed effect, and family relationship as random effect. [file 12916_2023_3149_MOESM6_ESM.docx]

**Additional file 7: Fig.S7**

**A B**


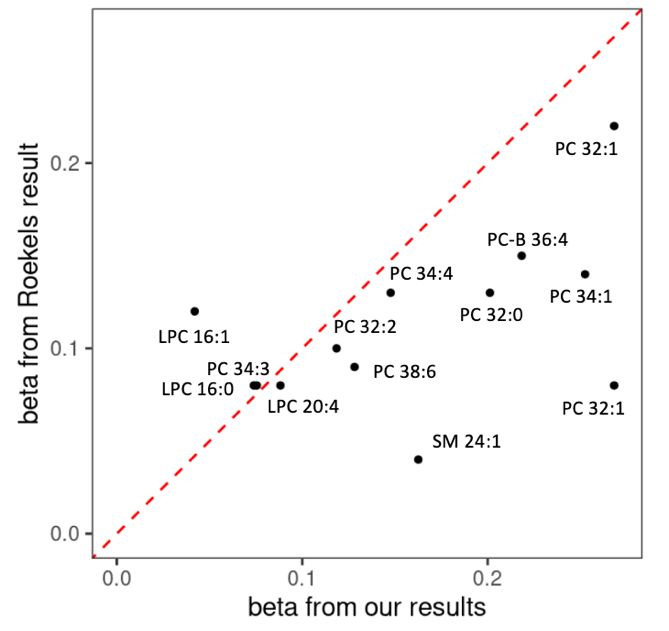

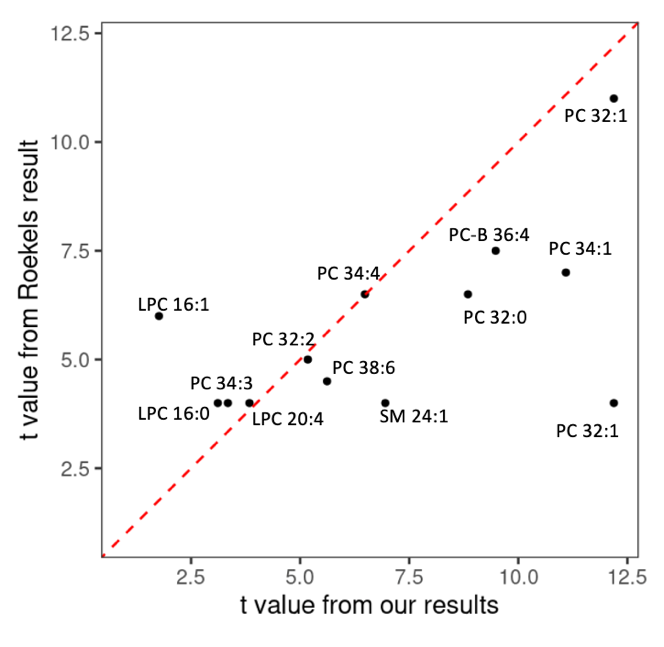

Supplement: Supplementary file 7 — Additional file 7: Fig. S7. Comparison to Roekel’s Study. Panel A: comparison of beta; Panel B: comparison of t value. Only in this analysis, alcohol consumption (g/day) in this study was plus 1 and natural log transformed. The sex and batch-adjusted residual of metabolites from linear mixed model were used as outcome. Then we applied linear mixed model for alcohol drinking and residual of metabolites in linear mixed model, adjusting for age, sex, smoking status, BMI, physical activity, diet score as fixed effect, and familial relationship as random effect. But Roekel’s study used linear model and covariates included age at blood collection, sex, country, fasting status at blood collection, smoking status, BMI, Cambridge physical activity index, and daily intake of energy, meat and meat products, fish, and shellfish. [file 12916_2023_3149_MOESM7_ESM.docx]
